# Statin use and cancer risk in the General Practice Research Database

**DOI:** 10.1038/sj.bjc.6601566

**Published:** 2004-02-03

**Authors:** J A Kaye, H Jick

**Affiliations:** 1 Boston Collaborative Drug Surveillance Program, Boston University School of Medicine, 11 Muzzey Street, Lexington, MA 02421, USA

**Keywords:** 3-hydroxy-3-methylglutaryl coenzyme A inhibitors, statins, hyperlipidaemia, epidemiology

## Abstract

In a matched case–control study using the General Practice Research Database, current statin use was not associated with a significantly altered risk of any of 13 studied cancers. Untreated hyperlipidaemia was associated with slightly increased risks of colon cancer (relative risk 1.8; 95% confidence interval 1.2–2.8), prostate cancer (1.5; 1.1–2.0), and bladder cancer (1.9; 1.2–3.1).

Inhibitors of 3-hydroxy-3-methylglutaryl coenzyme A reductase (‘statins’) inhibit the rate-limiting step in cholesterol synthesis. Rodent studies indicate that statins are carcinogenic (Newman and Hulley, 1996). Other evidence, however, suggests that statins may inhibit the growth of cancer cells by triggering apoptosis (Muller *et al*, 1998; Dimitroulakos *et al*, 2002; Wong *et al*, 2002), inhibiting angiogenesis (Weis *et al*, 2002; Park *et al*, 2002), or impairing the metastatic process (Alonso *et al*, 1998; Kusama *et al*, 2002). We undertook a matched case–control study of the relation between statin use and cancer risk.

## SUBJECTS AND METHODS

We carried out this study using information from the General Practice Research Database (GPRD). General practitioners enter patient data into computerised medical records and use the software to generate prescriptions, providing a complete record of medications (Jick *et al*, 1991; Jick *et al*, 2003). Cancer diagnoses are highly reliable when validated against consultants' letters and hospital discharge summaries (Jick *et al*, 1997), and age-specific time trends in the incidence of breast cancer, for example, are closely similar to those reported by the Office for National Statistics (Kaye *et al*, 2000).

### Base population

The base population comprised all subjects 50–89 years old who used antihyperlipidaemic drugs or had a recorded diagnosis of untreated hyperlipidaemia. We also included a random sample of 50 000 subjects of the same ages who neither used antihyperlipidaemic drugs nor had a recorded diagnosis of hyperlipidaemia. We excluded subjects diagnosed with any cancer other than the study cancer diagnoses (see below), and restricted our analysis to subjects who had at least 3 years of follow-up time in the GPRD (median 6.4, maximum 13.7).

### Cases and controls

We identified all subjects who had a first-time recorded diagnosis of any of 13 cancer diagnoses during 1990–2002. The study cancers were melanoma (79 cases) and carcinomas of the oesophagus (100), stomach (126), pancreas (125), colon (329), rectum (183), lung (605), prostate (569), kidney (39), bladder (227), breast (698), ovary (91), and endometrium (73). We identified up to five controls for each case matching on year of birth, sex, general practice, year of entry into the GPRD, and index date.

### Exposure

We analysed exposure to statins and nonstatins (fibrates, cholestyramine, colestipol, nicotinic acid, and acipimox). Subjects with a recorded diagnosis of hyperlipidaemia but no treatment composed a separate exposure category. We considered subjects to be current statin users if they received a prescription for a statin within the year before their index date and their first prescription was recorded more than a year before the index date. The median and maximum durations of statin use among current users were 29 and 124 months. The reference exposure group comprised subjects who had no history of hyperlipidaemia and no recorded use of antihyperlipidaemic drugs.

### Potential confounders

Body mass index was estimated based on each subject's first recorded weight within 1–5 years before the index date. Smoking was categorised as current, past, none, or unknown. Analyses were also adjusted for the average visit frequency during follow-up in the GPRD before the index date (0–1, 2–5, or 6+ visits per year).

### Statistical methods

We used conditional logistic regression to estimate the relative risk (odds ratio) for each of the study cancers in relation to exposure (PHREG procedure, SAS, version 8.02, SAS Institute Inc., Cary, NC, USA). We evaluated regression models including (1) only the main exposure variables conditional on the matching factors; (2) also smoking, body mass index, and average visit frequency; and (3) the exposure variables and whichever potential confounders were significantly associated with each cancer. We report results from the model that provided the best fit to the data for each cancer by likelihood ratio testing.

## RESULTS

We identified 3244 cases and 14 844 controls (Table 1

**Table 1:** Characteristics of cases and controls

	**Cases**		**Controls**	
	***N***	**%**	***N***	**%**
*Sex*				
Female	1585	48.9	7393	49.8
Male	1659	51.1	7451	50.2
				
*Age (years)*				
50–59	460	14.2	2206	14.9
60–69	1066	32.9	5156	34.7
70–79	1201	37.0	5508	37.1
80–89	517	15.9	1974	13.3
				
*Smoking*				
Smoker	668	20.6	2004	13.5
Ex-smoker	475	14.6	1809	12.2
Nonsmoker	1467	45.2	7763	52.3
Unknown	634	19.5	3268	22.0
				
*BMI* (*kg*/*m*^*2*^)				
<24	756	23.3	3026	20.4
24–28	851	26.2	4140	27.9
>28	675	20.8	3048	20.5
Unknown	962	29.7	4630	31.2

). Men and women were approximately equally represented. Current smoking was more prevalent among cases than controls.

### Current statin use

In all, 256 cases (7.9%) and 1066 controls (7.2%) were currently exposed to statins. Among current statin users, the overall relative risk for any study cancer was 1.0 (95% confidence interval 0.9–1.2) compared to the reference exposure group. For no specific cancer type was there a significantly altered relative risk among current statin users (Table 2

**Table 2:** Relative risk of specific cancers in relation to current statin use

	**Cases**		**Controls**			
	***N***	**%**	***N***	**%**	**RR**	**95% CI**
Cancer						
Oesophageal	9	9.0	34	7.9	0.8	0.3–1.8
Stomach	4	3.2	35	6.4	0.4	0.1–1.3
Colon	25	7.6	115	7.6	1.0	0.6–1.7
Rectal	23	12.6	59	7.0	1.6	0.9–2.8
Pancreatic	12	9.6	53	9.1	0.8	0.4–1.6
Lung	43	7.1	216	7.8	0.9	0.6–1.3
Melanoma	7	8.9	19	5.0	2.5	0.8–7.3
Breast	40	5.7	196	6.0	0.9	0.6–1.3
Endometrial	3	4.1	21	5.9	0.5	0.1–1.9
Ovarian	6	6.6	25	6.1	1.0	0.4–2.7
Prostate	62	10.9	204	8.2	1.3	1.0–1.9
Bladder	19	8.4	74	7.1	1.2	0.7–2.3
Kidney	3	7.7	15	8.0	1.0	0.3–4.2

a Current statin use is defined as at least one recorded prescription for a statin during the year before the index date (with or without current use of other antihyperlipidaemic drugs) with the first such prescription recorded at least 1 year before the index date.

). We did, however, find marginally significantly increased risks of 3.5 (1.1–10.9) and 4.2 (1.0–16.6) for colon cancer and rectal cancer among current statin users of 60 months or longer (estimates based on five and four exposed cases, respectively). However for both these cancers, the relative risks among current statin users with 24–59 months exposure (0.9 [0.4–1.8] and 1.4 [0.6–3.2]) were similar to those with less than 24 months duration (0.8 [0.4–1.9] and 1.2 [0.5–3.0], respectively).

### Current use of other antihyperlipidaemic drugs

For no specific cancer was there a significantly altered relative risk among current users of other antihyperlipidaemic drugs.

### Past use of statins and/or other antihyperlipidaemic drugs

Among 169 past statin users with no exposure to other antihyperlipidaemic drugs, 198 past users of other antihyperlipidaemic drugs with no exposure to statins, and 30 past users of both statins and other antihyperlipidaemic drugs, we observed increased relative risks for breast cancer (2.0; 1.2–3.1), endometrial cancer (5.3; 1.4–20.6), and ovarian cancer (5.5; 1.5–21.2). However, because the median duration of statin use among past statin users was 2 months (interquartile range <1–12 months), these results are not compatible with a biologically plausible association between past statin use and cancer risk.

### Untreated hyperlipidaemia

Among this group, we observed significantly increased risks of colon cancer (1.8; 1.2–2.8), prostate cancer (1.5; 1.1–2.0), and bladder cancer (1.9; 1.2–3.1).

## DISCUSSION

This study provides evidence that statin use is not associated with a substantially increased or decreased risk of the cancers we studied. However, we found that untreated hyperlipidaemia was associated with slightly increased risks of colon (1.8; 1.2–2.8), prostate (1.5; 1.1–2.0), and bladder cancer (1.9; 1.2–3.1).

Although the results of one randomised study suggested an increased risk of breast cancer associated with pravastatin use (Sacks *et al*, 1996), subsequent meta-analyses of clinical trials (Hebert *et al*, 1997; Bjerre and LeLorier, 2001; Pfeffer *et al*, 2002) have indicated that statin use is not associated with any increase in cancer risk.

In a nested case–control study using administrative databases in Montreal, Blais *et al* (2000) found a decreased risk of cancer (0.72; 0.57–0.92) among statin users compared with users of other lipid lowering drugs. As the base population included only users of lipid-lowering drugs, this study could not directly evaluate whether statins users have a higher or lower risk of cancer than nonusers. In another case–control study using data from interviews conducted in hospitals in four US metropolitan areas, Coogan *et al* (2002) found an increased risk of breast cancer (1.5; 1.0–2.3) among statin users compared with nonusers, but this was largely related to carcinoma *in situ* and may have been due to detection bias. In a study based on the Saskatchewan population, Beck *et al* (2003) found a slightly increased risk of breast cancer among older statin users (1.15, 0.97–1.37), but the association was more evident among short- than long-term users, arguing against a causal effect.

In summary, the data we present here are consistent with the hypothesis that statin use does not have a substantial effect on cancer risk. Longer follow-up of subjects in the GPRD is needed to determine whether these results remain consistent after more prolonged periods of statin treatment.
